# Synthesis, Characterization, and Thin-Film Transistor Response of Benzo[i]pentahelicene-3,6-dione

**DOI:** 10.3390/molecules27030863

**Published:** 2022-01-27

**Authors:** Maria Paola Bracciale, Guhyun Kwon, Dongil Ho, Choongik Kim, Maria Laura Santarelli, Assunta Marrocchi

**Affiliations:** 1 Department of Chemical Engineering Materials and Environment, University of Rome “Sapienza”, Via Eudossiana 18, 00184 Rome, Italy; marialaura.santarelli@uniroma1.it; 2 Department of Chemical and Biomolecular Engineering, Sogang University, Seoul 04107, Korea; kguhyun1119@nate.com (G.K.); hdnel@naver.com (D.H.); choongik@sogang.ac.kr (C.K.); 3 Department of Chemistry, Biology and Biotechnology, University of Perugia, Via Elce di Sotto 8, 06123 Perugia, Italy

**Keywords:** helicenes, organic thin film transistors, Diels-Alder reaction

## Abstract

Organic semiconductors hold the promise of simple, large area solution deposition, low thermal budgets as well as compatibility with flexible substrates, thus emerging as viable alternatives for cost-effective (opto)-electronic devices. In this study, we report the optimized synthesis and characterization of a helically shaped polycyclic aromatic compound, namely benzo[i]pentahelicene-3,6-dione, and explored its use in the fabrication of organic field effect transistors. In addition, we investigated its thermal, optical absorption, and electrochemical properties. Finally, the single crystal X-ray characterization is reported.

## 1. Introduction

The field of organic electronics has been pushed strongly forward over recent decades, due to the synergy between different disciplines, including materials science, physics, chemistry, and engineering, as well as the interplay between academic and industrial actors [1,2,3,4,5].

From a synthetic chemistry point of view, significant efforts have been made to develop organic semiconducting materials, which could offer a viable alternative to their traditional inorganic counterparts. A wide range of small molecules and polymers have been successfully designed and synthesized for applications in many different devices (e.g., organic thin-film transistors (OTFTs), electrooptic modulators, organic (OPVs) or hybrid solar cells, organic light-emitting diodes (OLEDs), laser diodes, optical waveguides, and sensors), including both p-type and n-type semiconductors [6,7,8]. The structural diversity in terms of the core type, side chains, substituents, bridging units, conjugation extent, and shape of organic semiconductors [9,10,11,12,13,14] enables the fine-tuning of the material’s properties depending on the specific device requirements. 

Among the molecular materials, the possibility to create devices from organic semiconductors with a helically shaped conjugated electronic system, namely helicenes, has been gaining attention recently.

Helicenes [15,16,17,18] are inherently dissymmetric structures, made up of ortho-fused aromatic or heteroaromatic rings, in which the repulsive steric overlap of the (terminal) aromatic units and, eventually, the presence of functional groups in them determines the non-planarity of the conjugated structure, imparting a helical twist. Hence, helicenes exist in both left-handed (M, minus) and right-handed (P, plus) chiral (i.e., enantiomeric) forms.

This helically chiral, fully conjugated architecture endows these molecules with unique chiroptical properties, e.g., high optical rotatory power, strong circularly polarized luminescence, and circular dichroism [19,20,21].

It has been demonstrated that helicenes may be able to self-assemble into long corkscrew-shaped columns, which subsequently aggregate to form large crystalline domains in both concentrated solutions and thin films [22,23,24,25]. Besides dramatically enhancing the helicene chiroptical properties, this supramolecular aggregation may provide unique charge transport properties, as observed for, e.g., DNA [26].

Despite their peculiar properties, however, helicenes still remain rather unexplored in solid-state (opto-)electronic devices. Racemic mixtures of tetrathia-[7]-helicene [27], naphthalene double [6]helicenes [28], pyrene double [4]heterohelicene [29], 5,14-diaryldiindeno[2,1-f:1′,2′-j]picene [30,31], perylene diimide double-[5]heterohelicene [32,33], and a multiple helicene containing two [5]helicene and four [4]helicene moieties [34] have been used in organic thin-film transistors (OTFTs). Some authors exploited the chirality of the helicenes by using the enantiomerically pure 1-aza[6]helicene [35] and perylene diimide double-[7]heterohelicene [36] in circularly polarized detecting OTFTs.

A handful of studies are available for other types of devices, such as organic light emitting diodes [37,38,39,40,41,42,43,44,45,46,47,48,49,50,51,52,53,54,55,56], organic solar cells [57,58,59], and hybrid DSSC [60] and perovskite solar cells [61,62,63,64,65,66,67,68,69,70,71,72].

In this paper, we report the optical, electrochemical, and structural characteristics of racemic benzo[i]pentahelicene-3,6-dione as well as its thin film, and OTFTs’ semiconductor properties.

## 2. Results and Discussion

### 2.1. Synthesis

**Scheme 1:**
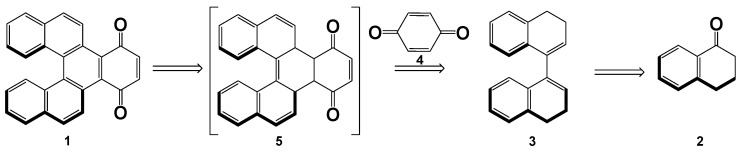
Retrosynthesis of [5]helicenebenzoquinone **1**.

Benzo[i]pentahelicene-3,6-dione (**1**, Scheme 1) was synthesized by optimizing a previously published procedure [73]. The simplicity in this design makes this system worthy of investigation. Indeed, many advanced semiconducting helical structures are extremely challenging to access and, therefore, small quantities of material are typically available for study. Basically, the synthesis of compound **1** involves the use of α-tetralone (**2**) as a starting material, which is subjected to self-coupling in the presence of a Zn-chlorotrimethylsilane system to afford 3,3′,4,4′-tetrahydro-l,l′-binaphthalene (**3**). The synthetic process was completed by the solvent-free Diels-Alder reaction of the diene **3** with ten-fold excess of 1,4-benzoquinone (**4**), followed by the in situ dehydrogenation of the resulting cycloadduct **5**. Notably, 1,4-benzoquinone, under these conditions, behaves simultaneously as a dienophile in the Diels-Alder reaction and as an oxidizing agent. Though the yield is just acceptable (~32%, see Materials and Methods), this one-step cycloaddition-dehydrogenation reaction is simple and may enable the production of the [5]helicenebenzoquinone **1** on a large scale, starting from readily available and reasonably cheap starting materials.

The Diels-Alder reaction represents a suitable and atom-efficient method for constructing annulated ring systems containing six-member rings [74], and it consequently offers an alternative synthetic approach to helicenes [75,76,77,78,79,80,81,82,83,84].

It is noteworthy to mention that, in our strategy, the use of excess 1,4-benzoquinone is not an issue, as the final optimized protocol also includes its recovery via steam distillation followed by filtration and reuse to minimize the waste associated with the process.

### 2.2. Thermal Properties

**Figure 1:**
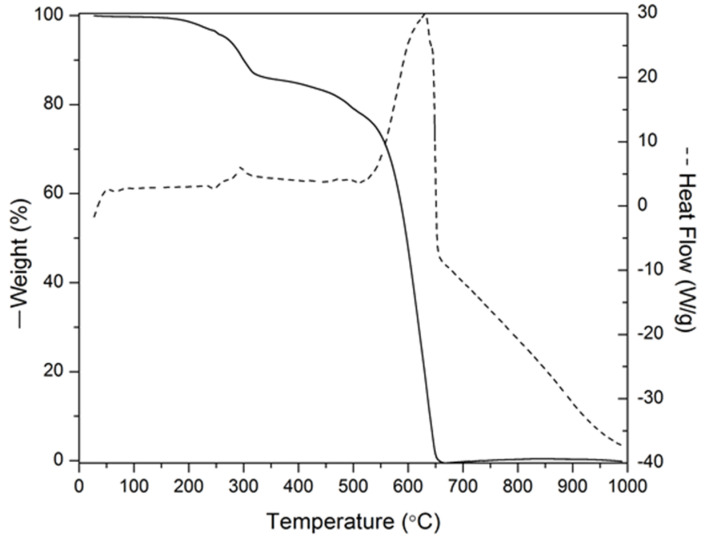
TGA (solid line) and DSC (dotted line) thermograms of the [5]helicenebenzoquinone (exotherm up).

Thermal stability and transitions for [5]helicenebenzoquinone were investigated by simultaneous TGA/DSC. Representative TGA/DSC analysis curves are reported in Figure 1. As shown, the thermal decomposition takes place in two main steps until 650 °C. In the first step, the quinone loses 13.9 wt% between 30 and 341 °C. In this temperature range, the DSC curve shows an endothermic peak at 247 °C, which could be assigned to a melting point, and an exothermic peak at 295 °C due to thermal degradation or evaporation process. In addition, a continual mass loss up to 650 °C (~86.5%) associated with a broad exothermic peak, appears at 631 °C in the DSC curve. As shown, the synthetized material featured remarkable thermal stability. The onset point of the weight loss with 5% weight loss temperature (decomposition temperature T_d_) was 268 °C, and no weight loss was observed at lower temperatures.

### 2.3. Optical and Electrochemical Properties

The solution and thin-film photophysical properties of the synthetized quinone were assessed by optical absorption (ultraviolet-visible (UV-vis)) spectroscopy, and cyclic voltammetry (CV) was used to identify its electrochemical behavior.

**Figure 2:**
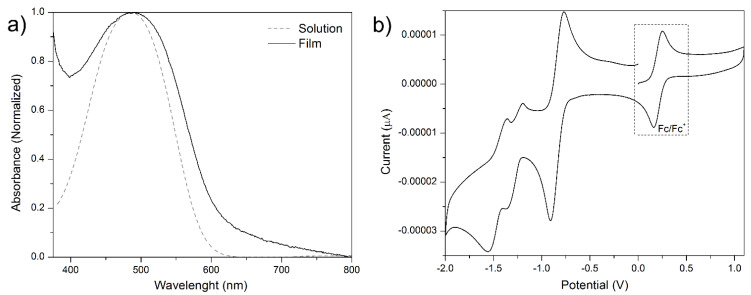
(**a**) UV-vis normalized absorptions of [5]helicenebenzoquinone in solution (CHCl_3_) and as a thin film; (**b**) cyclic voltammetry; 10^−3^ M CH_2_Cl_2_, 0.1 M NBu_4_PF_6_, T = 25 °C, scan rate: 100 mVs^−1^, reference electrode: Ag/AgCl, working electrode: glassy carbon. Fc/Fc^+^ was used as internal reference (E_1/2-ferrocene_ = 0.21 V).

Cyclic voltammogram and absorbance spectra of the compound are shown in Figure 2, and electrochemical and optical data are summarized in Table 1.

Optical absorption and emission maxima were performed in solution and as thin films on glass substrates (Figure 2a).

In chloroform (CHCl_3_), the studied quinone exhibits an absorption maximum (λ_max-sol_) located at 486 nm and a molar extinction coefficient (ε) of 8.33 × 10^3^ Lmol^−1^cm^−1^.

A thin film of the same compound shows a sizeable broadening and exhibits an absorption maximum (λ_max-film_) at 490 nm. The broadening and the small bathochromic shift (<10 nm), observed going from the solution to the thin film, is clear evidence of solid-state J-aggregate formation [85,86]. Interestingly, a tail in the [5]helicene film absorption profile can be observed, spanning from 600 to 800 nm. This might have a component [87] from spatially proximal interactions between the dipole–dipole interactions of the quinone fragment, facilitated by the tendency of the helicene molecules to self-assemble, that can modulate the spectral transition above 600 nm and create a tail in the absorption spectrum.

The band gap for the [5]helicene **1** was estimated from the low-energy band edges (Table 1). The optical band gaps of the compound in solution and solid state are estimated to be 2.11 and 2.01 eV, respectively.

The CV plot (Figure 2b) show the presence of a reversible reduction process with half-wave potentials at −1.46 V. No oxidation peaks are observed in the investigated electrochemical window.

From the reduction potential, LUMO and HOMO energies were estimated knowing that the SCE energy level is −4.8 eV below the vacuum level and the band gap obtained by optical absorption data (Eg_sol_) (Table 1), respectively. Using these relations, the LUMO value was estimated to lie at −3.14 eV, and HOMO was calculated as −5.25 eV.

### 2.4. Quantum Mechanical Calculations

**Figure 3:**
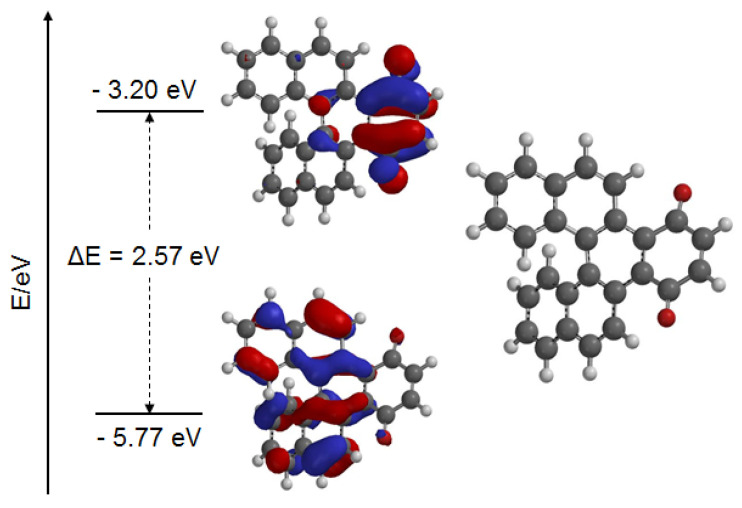
Computational (DFT) values of HOMO/LUMO/gap energies, MO electron density contours and structure of [5]helicenebenzoquinone.

To gain an insight into the electronic properties of [5]helicenebenzoquinone, quantum-chemical calculations were also performed. The optimized geometry, molecular orbitals’ contour plots (MO), and energy diagram are shown in Figure 3.

The molecular orbital (MO) energies and contours were calculated by using the DFT-optimized structure of the molecule [79]. Since charge is transported through the frontier molecular orbitals of organic semiconductors, their geometries, energies, and extents of delocalization directly affect carrier stability and intermolecular hopping rates. Ideally, the HOMO and LUMO are delocalized over the entire π-core of the semiconductor for p- and n-channel transport, respectively [86].

Here, the HOMO is delocalized over the ortho-anellated moiety, while the LUMO is more compact, since it is delocalized only on the benzoquinone subunit. This highly localized nature of the LUMO limits intermolecular π-overlap and may favor charge trapping at the quinone site. This may be a contributing factor to the limited mobility values of [5]helicenebenzoquinone (*vide infra*). This is not uncommon behavior [88] for quinone-containing semiconductors. The computed HOMO energy is −5.77 eV, while the LUMO energy is −3.20 eV.

The DFT-calculated HOMO and LUMO energies are in good agreement with the experimental values (Figure 3 and Table 1, respectively).

### 2.5. Crystallographic Characterization

The X-ray analysis was carried out on a recrystallized single crystal obtained from [5]helicenebenzoquinone solution. The crystal data and structure refinement are reported in Table 2 and Tables S1–S6 (Supplementary Information).

### 2.6. Thin-Film Transistor Characterization

**Figure 4:**
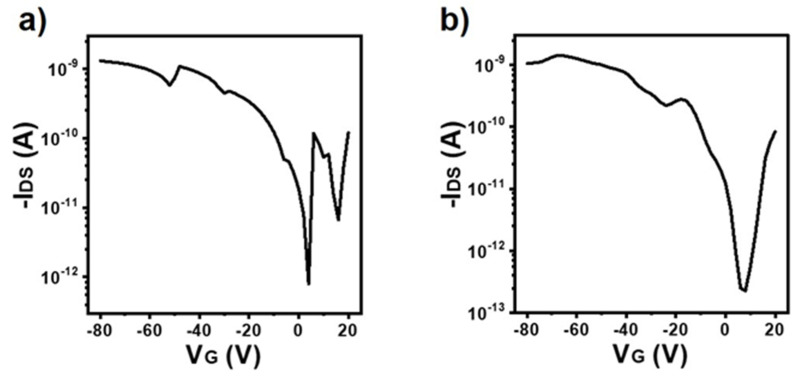
Transfer plot of the OTFT device obtained via (**a**) the SC method onto bare SiO_2_ (not annealed), and (**b**) the DC method onto the PS brush with annealing treatment (150 °C).

Top-contact OTFTs (1000 µm channel widths (W) and 50 µm channel lengths (L)) were fabricated by spin coating or drop casting films on 300 nm SiO_2_ (Bare), poly-4-vinylphenol (PVP) and PS-brush (PS-OH)-treated SiO_2_ substrates. The current–voltage characteristics of the fabricated organic thin film transistor (OTFT) devices were measured at room temperature and the device performance (mobility, current on/off ratio, and threshold voltage) data and representative output and transfer curves are reported in Table 3 and Figure 4, respectively. The field-effect carrier mobility (µ) of the semiconductor was calculated in the saturation regime using the conventional transistors relationship: µ_sat_ = (2I_DS_L)/[WC_ox_(V_sg_ − V_th_)^2^], where I_sd_ is the source-drain saturation current; C_ox_ is the areal capacitance value of the insulator layer, V_sg_ is the gate voltage, and V_th_ is the threshold voltage. The latter can be estimated as the x intercept of the linear section of the plot of V_sg_ vs. (I_sd_)^1/2^.

[5]Helicenebenzoquinone showed a p-type behavior, with hole mobilities of 10^−5^–10^−7^ cm^2^/Vs and current on/off ratio of 10^3^–10^4^.

The device response was found to be dependent on both the deposition method (SC or DC) and the dielectric surface treatment. Both have a critical impact on the π–π stacking and intermolecular interaction between the semiconductor molecules and influence the neighbouring molecular orbital overlapping. In the case of SC, although the uniformity of the thin film is generally excellent, there is a disadvantage of low crystallinity. On the other hand, DC generally results in highly crystalline thin films, whereas the uniformity is poor. Overall, devices fabricated by DC deposition performed better than the spin-coated counterparts. By comparing the performance of the SC and DC devices in Table 3, it can be implied that the slow, static process of DC facilitates preferable ordering of the [5]helicenebenzoquinone molecules compared to the SC method. The majority of the non-working and poor SC devices may be at least in part be attributed to the poor molecular ordering due to the accelerated solvent evaporation from spin-coating.

Among the devices obtained by the DC method, those on the PS brush demonstrated the best results. PS brush treatment is a widely used surface modification method which optimizes the morphology, surface energy, and viscoelastic properties of the resulting dielectrics [89]. The modification of the semiconductor/dielectric interface is crucial to the growth of the semiconductor layer, which leads to enhanced OTFT characteristics.

Representative micrographies of the thin films are reported in Figure S1 (Supplementary Information). It can be observed that in all cases they typically show gaps between (inhomogeneous) crystallites, which likely contribute to compromising charge transport. No evident correlation between T_D_ and device performance could be identified.

## 3. Materials and Methods

All chemicals were purchased from Merck KGaA, Darmstadt, Germany, and used without further purification unless otherwise noted. Column chromatography was performed on silica gel (230–400 mesh American Society for Testing and Materials, ASTM) as a stationary phase. 3,3’,4,4’-tetrahydro-l,1’-binaphthalene (**2**) was prepared according to a previously reported procedure [73].

NMR spectra were recorded on a Varian Associates (Palo Alto, CA, USA) VXR-400 multinuclear spectrometer using deuterated chloroform (CDCl_3_) as the solvent (internal Me_4_Si). Elemental microanalyses were performed using a Fisons’(Ipswich, London, UK) EA1106 CHN analyzer using atropine (C_17_H_23_NO_3_), 2,5-bis-2-(5-tertbutylbenzoxazol-yl)-thiophene (C_26_H_26_N_2_O_2_S; BBOT) and phenanthrene (C_14_H_10_) as a reference standard, with an accuracy of ≈ 2 μmol g^−1^. The melting point was measured on a Büchi 510 instrument (Büchi Labortechnik AG, Flawil, CH) and was not corrected. Thermogravimetric (TGA) and differential scanning calorimetric (DSC) analyses were performed on a TGA/DSC simultaneous apparatus (SDT Q600, TA Instruments, New Calstle, Delaware, UK) under a nitrogen flow rate of 100 mL min^−1^. Samples in an open platinum cell were heated from 30 up to 1000 °C with a rate of 10 °C min^−1^. Absorption spectra of the compound in chloroform (CHCl_3_) were recorded using a Varian Cary 100 UV-vis spectrophotometer (using a glass cuvette of 1 cm path length) over the spectral range of 350–800 nm. Electrochemical properties were determined using a Bass potentiostat, with a conventional three-electrode configuration (platinum-wire counter electrode, glassy carbon working electrode, and silver/silver chloride (Ag/AgCl) reference electrode). Cyclic voltammetry was performed in an electrolyte solution of 0.1 M tetrabutylammonium hexafluorophosphate (C_16_H_36_F_6_NP; TBAPF_6_) in dichloromethane (CH_2_Cl_2_; DCM) with scan rates between 50 and 100 mVs^−1^. The solutions were purged with nitrogen steam for about 15 min before every experiment. The glassy carbon working electrode was polished with a slurry of alumina and ultrapure water on a Buehler felt pad and rinsed thoroughly with ultrapure water. A ferrocene/ferrocenium redox (Fc/Fc+) couple was used as an internal standard. The HOMO and LUMO energy levels were calculated from the oxidation (E_ox_) and reduction (E_red_) potentials data using the empirical relation E_LUMO_ = [−(E_red_ − E_1/2-ferrocene_) + 4.8] eV or E_HOMO_ = [−(E_ox_ − E_1/2-ferrocene_) + 4.8] eV. Density functional theory (DFT) computations were carried out using the Spartan ’08 software package (Wavefunction, Inc., Irvine, CA, USA) at the B3LYP/6-31G* level. Single crystals of helicene were recrystallized by layering chloroform (CHCl_3_) solution of the synthetized material with hexane (C_6_H_14_). Suitable crystals were mounted in inert oil and transferred to the cold gas stream of a Bruker (Billerica, MA, USA) Kappa APEX CCD area detector equipped with a MoKα sealed tube with graphite. The crystal was kept at −173.16 °C during data collection. SADABS-2012/1 (Bruker) was used for absorption correction. Using Olex2 [90], the structure was solved with the XT structure solution program using direct methods and refined with the ShelXL [91] refinement package using least squares minimization.

### 3.1. Diels-Alder Reaction of 3,3’,4,4’-Tetrahydro-1,1’-binaphthalene (***3***) with 1,4-Benzoquinone (***4***)

The diene **3** (5.2 g, 19.9 mmol) and 1,4-benzoquinone (**4**) (22 g, 0.2 mol) were heated together at 150–170 °C for 18 h in a degassed flask. The excess benzoquinone was removed by steam distillation from the crude reaction mixture, and the residue was purified by column chromatography (silica gel, hexane/ethylacetate 3:1) to afford benzo[i]pentahelicene-3,6-dione (**1**). Yield: 32%; m.p. 246–247 °C. The spectra matched those in the literature. ^1^H-NMR (CDCl_3_) δ 6.97 (s, 1H), 7.21 (dd, 1H, J = 8.6, 7.0 Hz), 7.54 (dd, 1H, J = 8.1, 7.0 Hz), 7.92 (d, 1H, J = 8.1 Hz), 8.04 (d, 1H, J = 9.2 Hz), 8.13 (d, 1H, J = 8.6 Hz), 9.24 (d, 1H, J = 9.2 Hz). An. Calcd. for C_26_H_14_O_2_: C%, 87.13; H%, 3.94. Found: C%, 87.30; H%, 3.96.

### 3.2. OTFTs Fabrication and Characterization

Bottom-gate/top-contact organic thin-film transistors (OTFTs) were fabricated on heavily n-doped (100) silicon substrate (resistivity < 0.005 Ωcm) with a thermally grown silicon dioxide (300 nm SiO_2_) as the dielectric layer. Prior to deposition, the Si/SiO_2_ substrate were cleaned in an ultrasonic bath using acetone (C_3_H_6_O) for 15 min, dried using a nitrogen gun, and cleaned with air plasma for 5 min (Harrick Plasma, Ithaca, NY, USA18 W). In order to reduce charge trapping and thus enhance mobility, the SiO_2_ substrate was modified with poly-4-vinylphenol ((C_8_H_8_O)_n_; PVP) or PS-brush ((CH_2_CH(C_6_H_5_))_n_OH; PS-OH). Semiconducting layers were deposited via two different solution-processing methods: spin-coating (SC) and drop-casting (DC). For the SC process, the pentahelicenebenzoquinone was dissolved in chlorobenzene (C_6_H_5_Cl), dichlorobenzene (C_6_H_4_Cl_2_), or toluene (C_7_H_8_), and the solutions (15 mg/mL) were spin-coated at 3000 rpm for 30 s onto the substrates. The substrates were annealed at 150 °C under vacuum overnight. For the DC process, a 0.2 wt% solution of semiconductor in chlorobenzene (C_6_H_5_Cl) was deposited onto the substrates heated at 100 and 60–70 °C for bare, PS-brush- and PVP-treated substrates, respectively. The substrates were then annealed at 130–150 °C for 5 h under vacuum. Top contacts were fabricated by vapor deposition of gold electrodes (~10^−6^ Torr, 0.2 Ås^−1^, ~40 nm thick) onto the semiconductor thin films through a shadow mask to obtain devices with a channel width (W) and length (L) of 1000 and 50 μm, respectively. The capacitance of the 300 nm SiO_2_ gate insulator was 11.4 nFcm^−2^. Characterization of the devices was performed at room temperature in ambient atmosphere using a Keithley 4200 SCS (Keithley Instruments, Cleveland, OH, USA).

## 4. Conclusions

A helicene quinone, namely benzo[i]pentahelicene-3,6-dione, was prepared in one-pot reaction conditions via Diels–Alder cycloaddition-dehydrogenation steps. Its thermal, optical, and electrochemical behavior, along with its single-crystal structure and electronic structure, were also reported. [5]Helicenebenzoquinone exhibited excellent thermal stability, and near UV-vis electronic absorption.

An OTFT that incorporated the helical compound as a semiconductor with the configuration of bottom-gate/top-contact attained hole mobilities in the range of 10−5–10−7 cm2/Vs and current on/off ratio of 103–104 was formulated. The carrier mobilities were found to depend on the thin-film deposition method, and surface treatments.

This study contributes to gaining insights into the structure–properties–device response relationship in the class of helically shaped semiconducting materials. The strongly localized LUMO may be at least partially responsible for the limited mobility values of [5]helicenebenzoquinone. The tendency to form J-aggregates (i.e., less orbital overlap) and the presence of significant gaps between (heterogeneous) crystallites in thin-films also possibly compromise charge transport.

On the other hand, the LUMO localization and the J-aggregation, along with the excellent thermal stability, suggest the use of [5]helicenequinone in OLED devices with thermally activated delayed fluorescence (TADF) emitters [92].

Hence, future developments will be focused on expanding the range of device applications, including organic–inorganic hybrids. This is also worth pursuing in view of the fact that the optimized synthetic method enables us to prepare the [5]helicenebenzoquinone on a large scale, which is a key requirement for studying organics’ applications as materials for electronics. In addition, its structural simplicity, compared with other advanced double or multiple helicenes, is a design principle that can be applied in the development of semiconductors, to make the leap beyond the laboratory scale.

New helical architectures will be realized by introducing, e.g., substituents at the terminal aromatic rings, to tune the helical twisting of the structure, which can ultimately result in unique stacking structures.

Their optical resolution into enantiomerically pure forms will be also pursued.

## Figures and Tables

**Table 1:** Thermal, electrochemical and spectroscopic characterization of [5]helicenebenzoquinone **1**.

m.p (°C)	E_1/2-ox_ (V)	E_1/2-red_ (V)	λ_max__-sol_ (nm) ^a^	ε (Lmol^−1^cm^−^^1^)	Eg_sol_ (eV) ^a^	λ_max__-film_ (nm) ^b^	Eg_film_ (eV) ^b^	LUMO (eV) ^c^	HOMO (eV) ^d^
246	-	−1.67	486	8.33 · 10^3^	2.11	490	2.01	−3.14	−5.25

^a^ Optical band gap estimated from the low-energy band edge in the optical spectrum. Concentrations are 10^−4^–10^−5^ M in CHCl_3_; ^b^ Optical bandgap estimated from absorption onset (Eg = 1240/λ_onset_) of spin-cast film from 5 mg/mL of CHCl_3_ solution on glass substrates; ^c^ E_LUMO_ = [−(E_red_ − E_1/2-ferrocene_) + 4.8]; ^d^ E_HOMO_ = E_LUMO_ − E_opt.gap_.

**Table 2:** Summary of crystal data and structure refinement.

Parameter	Data
Empirical Formula	C_26_H_14_O_2_
Formula weight	358.37
Temperature/°C	−173.16
Crystal system	Orthorhombic
Space group	Pbca (no. 61)
a /Å, b/Å, c/Å	12.1651 (3), 7.4140 (2), 36.6996 (7)
α/°, β/°, γ/°	90, 90, 90
Volume / Å^3^	3310.01 (14)
Z	8
ρ_calc_/mg mm^−3^	1.438
μ(MoKα)/mm^−1^	0.090
F (000)	1488
Crystal size/mm^3^	0.24 × 0.15 × 0.069
2Θ range for data collection	5.562 to 56.558°
Index ranges	−15 ≤ h ≤ 16, −9 ≤ k ≤ 9, −48 ≤ l ≤ 48
Reflections collected	55,869
Independent reflections	4094 [R(int) = 0.1036]
Data/restraints/parameters	4094/0/253
Goodness-of-fit on F^2^	1.035
Final R indexes [I > 2σ (I)]	R_1_ = 0.0459, wR_2_ = 0.1030
Final R indexes [all data]	R_1_ = 0.0698, wR_2_ = 0.1151
Largest diff. peak/hole/e Å^−3^	0.301/−0.228

**Table 3:** Hole mobilities (cm^2^/V^−1^s^−1^), current on/off ratios (I_on_/I_off_), and threshold voltages (V_T_, V) for films deposited at various substrates, substrate temperatures (T_D,_ °C) and annealing temperatures (T_A_, °C); nw: not working.

Substrate	Solvent	T_D_	T_A_	µ_h_	I_on_/I_off_	V_T_
Spin-Coated (SC)						
Bare	chlorobenzene	-	-	1.6 × 10^−5^	1.5 × 10^3^	1.5
Bare	dichlorobenzene	-	150	nw		
Bare	Toluene	-	150	nw		
PVP	dichlorobenzene	-	150	nw		
PVP	Toluene	-	150	nw		
PS brush	dichlorobenzene	-	150	nw		
PS brush	Toluene	-	150	nw		
**Drop-casted (DC)**						
Bare	chlorobenzene	100	130	1.5 × 10^−7^	3.0 × 10^4^	−23
PVP	chlorobenzene	100	130	5.3 × 10^−5^	6.0 × 10^3^	−10
PS brush	dichlorobenzene	60	150	6.0 × 10^−5^	3.0 × 10^3^	−67
PS brush	chlorobenzene	70	-	2.5 × 10^−6^	5.0 × 10^3^	−35
PS brush	chlorobenzene	70	150	4.8 × 10^−5^	2.5 × 10^4^	−23

## Supplementary Materials

The following supporting information can be downloaded online, Figure S1: Microscopic view of thin films obtained by (a) SC from chlorobenzene onto bare SiO_2_ (not annealed); (b) SC from dichlorobenzene onto bare SiO_2_ (annealed at 150 °C); (c) SC from dichlorobenzene onto bare PVP (annealed at 150 °C); (d) DC from chlorobenzene onto bare SiO_2_ (annealed at 130 °C; T_D_ = 100 °C) and (e) onto PS brush (annealed at 150 °C; T_D_ = 70 °C); Table S1: Fractional Atomic Coordinates (×10^4^) and Equivalent Isotropic Displacement Parameters (Å^2^ × 10^3^) for compound **1**.; Table S2: Anisotropic Displacement Parameters (Å^2^ × 10^3^) for compound **1**.; Table S3: Bond Lengths for compound **1**.; Table S4: Bond Angles for compound **1**.; Table S5: Torsion Angles for compound **1**; Table S6: Hydrogen Atom Coordinates (Å × 10^4^) and Isotropic Displacement Parameters (Å^2^ × 10^3^) for compound **1**.

## Sample Availability

Samples of the compounds are not available from the authors.
