# Supplementary material for: Synthesis, Characterization, and Thin-Film Transistor Response of Benzo[i]pentahelicene-3,6-dione
Source: Molecules. 2022 Jan 27;27(3):863. doi: 10.3390/molecules27030863 (PMC8840029; doi:10.3390/molecules27030863)
Supplement: Supplementary file 1 [file molecules-27-00863-s001.zip › molecules-1558047-supplementary.pdf]

Synthesis, characterization, and thin-film transistor response of benzo[i]pentahelicene-3,6-dione

Maria Paola Bracciale[1,*], Guhyun Kwon,[2] Dongil Ho,[2] Choongik Kim,[2] Maria Laura Santarelli,[1] and Assunta Marrocchi[3,*]

Department of Chemical Engineering Materials and Environment, University of Rome "Sapienza", Via Eudossiana 18, 00184 Rome, Italy; mariapaola.bracciale@uniroma1.it (M.P.B.); marialaura.santarelli@uniroma1.it (M.L.S.)
Department of Chemical and Biomolecular Engineering, Sogang University, Seoul 04107, Korea; choongik@sogang.ac.kr (C.K.); hdnel@naver.com (D. H.) kguhyun1119@nate.com (G. K.)
Department of Chemistry, Biology and Biotechnology University of Perugia, Via Elce di Sotto 8, 06123 Perugia, Italy; assunta.marrocchi@unipg.it (A.M.)

Table S1 Fractional Atomic Coordinates (×10^4) and Equivalent Isotropic Displacement Parameters (Å^2×10^3) for compound **1**. U_{eq} is defined as 1/3 of of the trace of the orthogonalised U_{IJ} tensor.

**Atom**	*x*	*y*	*z*	**U(eq)**
O1	5087.5(9)	-650.0(16)	1132.6(3)	21.0(3)
O2	7606.6(9)	2483.8(17)	139.0(3)	23.5(3)
C1	6871.3(12)	548(2)	1003.2(4)	12.9(3)
C2	5682.2(12)	180(2)	923.2(4)	15.8(3)
C3	5245.5(13)	726(2)	566.1(5)	20.7(3)
C4	5896.8(13)	1347(2)	305.3(4)	20.2(3)
C5	7082.8(13)	1628(2)	366.6(4)	16.4(3)
C6	7572.1(12)	978(2)	715.9(4)	13.0(3)
C7	8737.5(12)	761.4(19)	761.4(4)	13.1(3)
C8	9496.5(13)	903(2)	462.8(4)	15.7(3)
C9	10569.9(13)	443(2)	501.8(4)	17.3(3)
C10	10963.4(12)	-344(2)	829.3(4)	15.4(3)
C11	12041.2(13)	-1056(2)	853.0(4)	20.3(3)
C12	12395.1(13)	-1904(2)	1160.5(5)	22.6(4)
C13	11666.9(13)	-2158(2)	1452.1(4)	20.8(3)
C14	10619.1(12)	-1469(2)	1439.3(4)	16.3(3)
C15	10249.0(11)	-475(2)	1134.1(4)	13.2(3)
C16	9151.2(11)	284.7(19)	1109.5(4)	12.0(3)
C17	8449.9(12)	540(2)	1421.6(4)	12.4(3)
C18	8860.6(12)	999(2)	1782.5(4)	13.6(3)
C19	9921.3(13)	1721(2)	1843.2(4)	16.8(3)
C20	10254.6(13)	2241(2)	2184.0(4)	20.3(3)
C21	9549.5(14)	2051(2)	2484.9(4)	24.1(4)
C22	8508.3(14)	1415(2)	2434.1(4)	21.7(4)
C23	8130.4(13)	940(2)	2082.4(4)	16.5(3)
C24	7000.0(13)	558(2)	2021.4(4)	17.8(3)
C25	6589.5(12)	410(2)	1680.3(4)	16.2(3)
C26	7298.3(11)	450(2)	1367.3(4)	13.0(3)

Table S2 Anisotropic Displacement Parameters (Å^2×10^3) for compound **1**. The Anisotropic displacement factor exponent takes the form: $-2\pi^2[h^2a^{*2}U_{11}+...+2hka\times b\times U_{12}]$

**Atom**	**U$_{11}$**	**U$_{22}$**	**U$_{33}$**	**U$_{23}$**	**U$_{13}$**	**U$_{12}$**
O1	14.6(5)	20.9(6)	27.5(6)	-1.0(5)	2.2(5)	-2.1(5)
O2	26.0(6)	26.7(6)	17.7(5)	6.5(5)	-2.0(5)	0.4(5)
C1	12.8(7)	9.4(7)	16.4(7)	-1.1(6)	-0.8(5)	1.0(6)
C2	13.0(7)	11.3(7)	23.0(7)	-3.0(6)	-0.2(6)	2.3(6)
C3	15.7(7)	18.6(8)	27.8(8)	-2.0(7)	-7.8(6)	1.4(6)
C4	21.8(8)	17.8(8)	21.1(8)	0.6(6)	-9.7(6)	2.2(6)
C5	21.1(8)	13.0(7)	15.2(7)	-2.2(6)	-2.4(6)	2.6(6)
C6	15.2(7)	10.4(7)	13.5(7)	-1.8(6)	-1.3(6)	-0.8(5)
C7	14.6(7)	11.1(7)	13.6(7)	-1.4(5)	0.2(6)	-1.8(5)
C8	18.3(7)	17.4(8)	11.4(7)	-0.3(6)	0.5(6)	-3.2(6)
C9	18.9(7)	18.0(8)	15.0(7)	-2.8(6)	5.7(6)	-4.5(6)
C10	13.1(7)	15.6(7)	17.6(7)	-4.9(6)	0.9(5)	-3.5(6)
C11	13.4(7)	22.2(8)	25.1(8)	-7.1(7)	5.0(6)	-2.1(6)
C12	13.9(7)	21.9(9)	31.9(9)	-8.8(7)	-2.9(7)	4.1(6)
C13	20.6(8)	18.1(8)	23.6(8)	-2.0(6)	-5.9(6)	4.0(6)
C14	16.6(7)	14.1(7)	18.1(7)	-2.1(6)	-0.3(6)	0.3(6)
C15	12.0(7)	12.4(7)	15.2(7)	-3.5(6)	0.3(5)	-2.8(6)
C16	12.5(7)	9.8(7)	13.7(7)	0.2(5)	0.1(5)	-2.0(5)
C17	15.4(7)	9.9(7)	11.9(7)	0.4(5)	0.3(5)	-0.2(5)
C18	17.6(7)	11.2(7)	11.9(7)	0.2(5)	-0.8(5)	3.4(6)
C19	18.6(7)	15.2(7)	16.5(7)	-1.3(6)	-1.5(6)	3.0(6)
C20	20.5(8)	19.7(8)	20.7(8)	-3.5(7)	-6.3(6)	3.9(7)
C21	30.6(9)	27.3(9)	14.5(7)	-5.1(7)	-6.8(6)	10.6(7)
C22	28.5(9)	24.8(9)	11.8(7)	-0.3(6)	2.0(6)	8.0(7)
C23	21.5(8)	14.3(7)	13.8(7)	1.4(6)	0.9(6)	3.5(6)
C24	21.0(8)	15.4(8)	17.0(7)	0.9(6)	8.1(6)	2.8(6)
C25	14.4(7)	14.2(7)	20.0(7)	0.3(6)	4.1(6)	0.8(6)
C26	14.3(7)	8.9(7)	15.7(7)	-0.6(6)	0.9(5)	0.4(5)

Table S3 Bond Lengths for compound **1**.

Atom	Atom	Length/Å	Atom	Atom	Length/Å
O1	C2	1.2218(19)	C12	C13	1.402(2)
O2	C5	1.2272(19)	C13	C14	1.374(2)
C1	C2	1.501(2)	C14	C15	1.414(2)
C1	C6	1.393(2)	C15	C16	1.452(2)
C1	C26	1.4355(19)	C16	C17	1.4406(19)
C2	C3	1.471(2)	C17	C18	1.4561(19)
C3	C4	1.325(2)	C17	C26	1.416(2)
C4	C5	1.475(2)	C18	C19	1.414(2)
C5	C6	1.493(2)	C18	C23	1.415(2)
C6	C7	1.437(2)	C19	C20	1.370(2)
C7	C8	1.437(2)	C20	C21	1.405(2)
C7	C16	1.4179(19)	C21	C22	1.364(2)
C8	C9	1.357(2)	C22	C23	1.415(2)
C9	C10	1.419(2)	C23	C24	1.422(2)
C10	C11	1.416(2)	C24	C25	1.352(2)
C10	C15	1.420(2)	C25	C26	1.4369(19)
C11	C12	1.362(2)			

Table S4 Bond Angles for compound **1**.

Atom	Atom	Atom	Angle/°	Atom	Atom	Atom	Angle/°
C6	C1	C2	118.91(13)	C13	C14	C15	121.06(14)
C6	C1	C26	119.65(13)	C10	C15	C16	119.17(13)
C26	C1	C2	121.44(13)	C14	C15	C10	117.71(13)
O1	C2	C1	122.63(14)	C14	C15	C16	122.95(13)
O1	C2	C3	119.02(14)	C7	C16	C15	118.62(13)
C3	C2	C1	118.18(13)	C7	C16	C17	118.25(13)
C4	C3	C2	121.54(14)	C17	C16	C15	123.11(13)
C3	C4	C5	121.60(14)	C16	C17	C18	123.41(13)
O2	C5	C4	118.49(14)	C26	C17	C16	117.89(13)
O2	C5	C6	122.97(14)	C26	C17	C18	118.58(13)
C4	C5	C6	118.38(13)	C19	C18	C17	123.01(13)
C1	C6	C5	118.66(13)	C19	C18	C23	117.51(13)
C1	C6	C7	119.38(13)	C23	C18	C17	119.00(13)
C7	C6	C5	121.95(13)	C20	C19	C18	121.34(15)
C6	C7	C8	122.51(13)	C19	C20	C21	120.56(15)
C16	C7	C6	118.87(13)	C22	C21	C20	119.59(14)
C16	C7	C8	118.51(13)	C21	C22	C23	120.83(15)
C9	C8	C7	121.31(14)	C18	C23	C24	119.40(14)
C8	C9	C10	121.13(14)	C22	C23	C18	119.86(14)
C9	C10	C15	119.27(13)	C22	C23	C24	120.50(14)
C11	C10	C9	121.16(14)	C25	C24	C23	121.28(14)
C11	C10	C15	119.52(14)	C24	C25	C26	121.12(14)
C12	C11	C10	121.05(15)	C1	C26	C25	121.88(13)
C11	C12	C13	119.65(15)	C17	C26	C1	119.11(13)
C14	C13	C12	120.69(15)	C17	C26	C25	118.81(13)

Table S5 Torsion Angles for compound **1**.

A	B	C	D	Angle/°
O1	C2	C3	C4	-168.27(16)
O2	C5	C6	C1	159.65(15)
O2	C5	C6	C7	-21.3(2)
C1	C2	C3	C4	7.2(2)
C1	C6	C7	C8	170.00(14)
C1	C6	C7	C16	-6.2(2)
C2	C1	C6	C5	19.2(2)
C2	C1	C6	C7	-159.90(13)
C2	C1	C26	C17	170.55(13)
C2	C1	C26	C25	-14.5(2)
C2	C3	C4	C5	-3.7(2)
C3	C4	C5	O2	-167.83(16)
C3	C4	C5	C6	7.6(2)
C4	C5	C6	C1	-15.6(2)
C4	C5	C6	C7	163.51(14)
C5	C6	C7	C8	-9.1(2)
C5	C6	C7	C16	174.75(13)
C6	C1	C2	O1	160.08(15)
C6	C1	C2	C3	-15.2(2)
C6	C1	C26	C17	-8.9(2)
C6	C1	C26	C25	166.02(14)
C6	C7	C8	C9	-170.53(14)
C6	C7	C16	C15	161.00(13)
C6	C7	C16	C17	-17.6(2)
C7	C8	C9	C10	5.6(2)
C7	C16	C17	C18	-147.85(14)
C7	C16	C17	C26	28.0(2)
C8	C7	C16	C15	-15.3(2)
C8	C7	C16	C17	166.12(13)
C8	C9	C10	C11	170.99(15)
C8	C9	C10	C15	-6.8(2)
C9	C10	C11	C12	-176.13(15)
C9	C10	C15	C14	172.12(14)
C9	C10	C15	C16	-3.2(2)
C10	C11	C12	C13	3.1(2)
C10	C15	C16	C7	14.2(2)
C10	C15	C16	C17	-167.32(13)
C11	C10	C15	C14	-5.7(2)
C11	C10	C15	C16	178.98(14)
C11	C12	C13	C14	-3.7(2)
C12	C13	C14	C15	-0.5(2)
C13	C14	C15	C10	5.2(2)
C13	C14	C15	C16	-179.69(14)
C14	C15	C16	C7	-160.89(14)
C14	C15	C16	C17	17.6(2)
C15	C10	C11	C12	1.6(2)
C15	C16	C17	C18	33.7(2)
C15	C16	C17	C26	-150.52(14)
C16	C7	C8	C9	5.6(2)
C16	C17	C18	C19	18.1(2)
C16	C17	C18	C23	-170.05(14)
C16	C17	C26	C1	-14.8(2)
C16	C17	C26	C25	170.13(13)
C17	C18	C19	C20	175.91(15)
C17	C18	C23	C22	-178.76(14)
C17	C18	C23	C24	-4.4(2)
C18	C17	C26	C1	161.23(13)
C18	C17	C26	C25	-13.8(2)
C18	C19	C20	C21	0.7(2)
C18	C23	C24	C25	-6.0(2)
C19	C18	C23	C22	-6.5(2)
C19	C18	C23	C24	167.93(14)
C19	C20	C21	C22	-2.8(2)
C20	C21	C22	C23	0.2(3)
C21	C22	C23	C18	4.5(2)
C21	C22	C23	C24	-169.78(16)
C22	C23	C24	C25	168.38(15)
C23	C18	C19	C20	3.9(2)
C23	C24	C25	C26	6.4(2)
C24	C25	C26	C1	-171.15(15)
C24	C25	C26	C17	3.8(2)
C26	C1	C2	O1	-19.4(2)
C26	C1	C2	C3	165.37(14)
C26	C1	C6	C5	-161.32(13)
C26	C1	C6	C7	19.6(2)
C26	C17	C18	C19	-157.71(14)
C26	C17	C18	C23	14.1(2)

Table S6 Hydrogen Atom Coordinates (Å×10^4) and Isotropic Displacement Parameters (Å^2×10^3) for compound **1**.

**Atom**	*x*	*y*	*z*	**U(eq)**
H3	4478	631	521	25
H4	5593	1619	73	24
H8	9242	1326	234	19
H9	11066	652	307	21
H11	12525	-939	651	24
H12	13132	-2322	1177	27
H13	11900	-2812	1661	25
H14	10135	-1663	1639	20
H19	10412	1847	1644	20
H20	10969	2733	2217	24
H21	9796	2364	2722	29
H22	8031	1290	2637	26
H24	6524	405	2224	21
H25	5819	277	1647	19

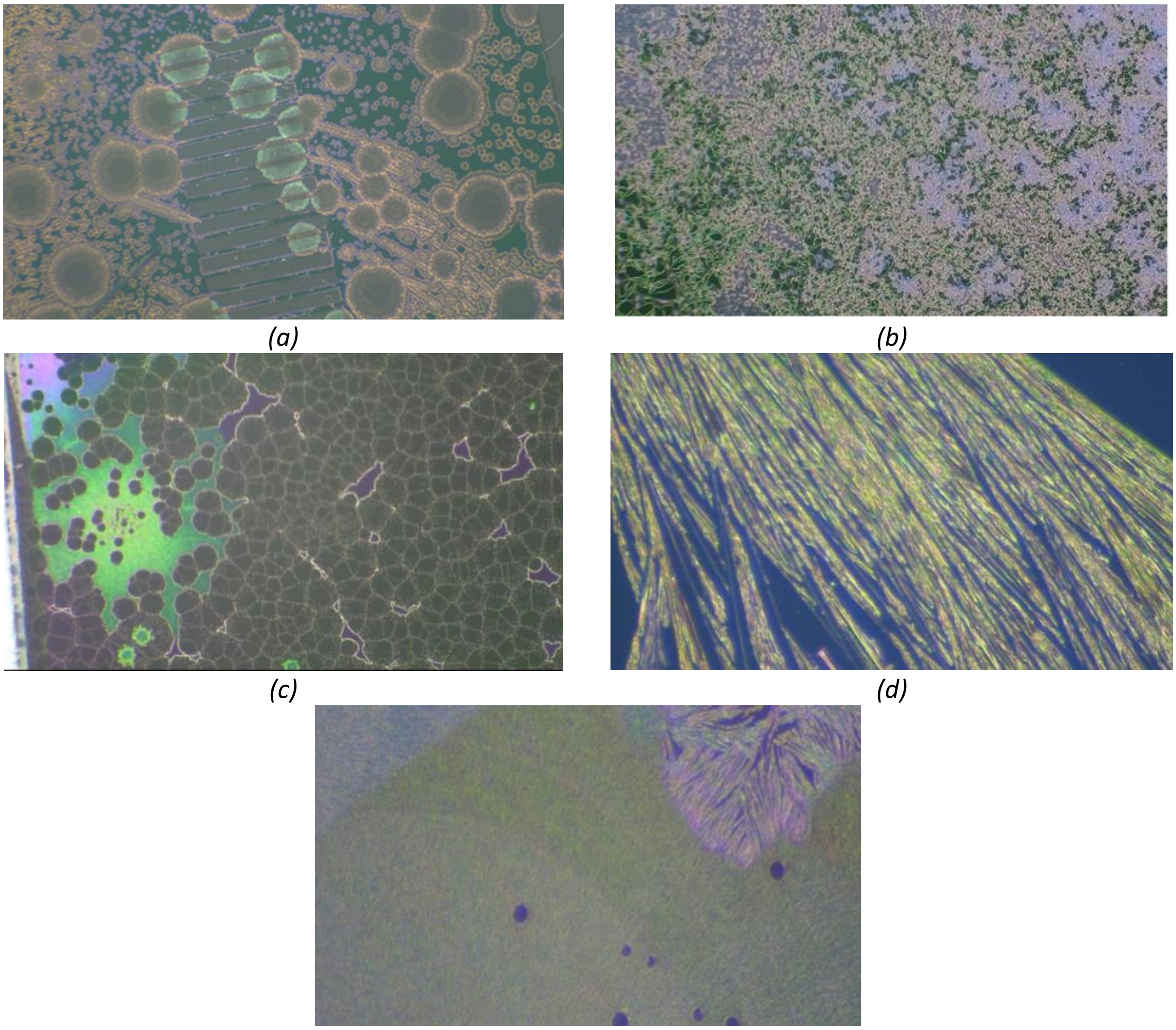

(e)

Figure S1. Microscopic view of thin films obtained by (a) SC from chlorobenzene onto bare SiO_2 (not annealed); (b) SC from dichlorobenzene onto bare SiO_2 (annealed at 150°C); (c) SC from dichlorobenzene onto bare PVP (annealed at 150°C); (d) DC from chlorobenzene onto bare SiO_2 (annealed at 130°C; T_D= 100°C) and (e) onto PS brush (annealed at 150°C; T_D= 70°C)
